# Using C‐Arm X‐ray images from marker insertion to confirm the gold fiducial marker identification in an MRI‐only prostate radiotherapy workflow

**DOI:** 10.1002/acm2.12478

**Published:** 2018-10-24

**Authors:** Christian Gustafsson, Emilia Persson, Adalsteinn Gunnlaugsson, Lars E. Olsson

**Affiliations:** ^1^ Department of Hematology, Oncology and Radiation Physics Skåne University Hospital Lund Sweden; ^2^ Department of Translational Medicine Medical Radiation Physics Lund University Malmö Sweden

**Keywords:** gold fiducial markers, MRI‐only prostate, MRI‐only radiotherapy, synthetic CT

## Abstract

Prostate cancer radiotherapy workflows, solely based on magnetic resonance imaging (MRI), are now in clinical use. In these workflows, intraprostatic gold fiducial markers (GFM) show similar signal behavior as calcifications and bleeding in T2‐weighted MRI‐images. Accurate GFM identification in MRI‐only radiotherapy workflows is therefore a major challenge. C‐arm X‐ray images (CkV‐images), acquired at GFM implantation, could provide GFM position information and be used to confirm correct identification in T2‐weighted MRI‐images. This would require negligible GFM migration between implantation and MRI‐imaging. Marker migration was therefore investigated. The aim of this study was to show the feasibility of using CkV‐images to confirm GFM identification in an MRI‐only prostate radiotherapy workflow. An anterior‐posterior digitally reconstructed radiograph (DRR)‐image and a mirrored posterior‐anterior CkV‐image were acquired two weeks apart for 16 patients in an MRI‐only radiotherapy workflow. The DRR‐image originated from synthetic CT‐images (created from MRI‐images). A common image geometry was defined between the DRR‐ and CkV‐image for each patient. A rigid registration between the GFM center of mass (CoM) coordinates was performed and the distance between each of the GFM in the DRR‐ and registered CkV‐image was calculated. The same methodology was used to assess GFM migration for 31 patients in a CT‐based radiotherapy workflow. The distance calculated was considered a measure of GFM migration. A statistical test was performed to assess any difference between the cohorts. The mean absolute distance difference for the GFM CoM between the DRR‐ and CkV‐image in the MRI‐only cohort was 1.7 ± 1.4 mm. The mean GFM migration was 1.2 ± 0.7 mm. No significant difference between the measured total distances of the two cohorts could be detected (*P* = 0.37). This demonstrated that, a C‐Arm X‐ray image acquired from the GFM implantation procedure could be used to confirm GFM identification from MRI‐images. GFM migration was present but did not constitute a problem.

## 1 INTRODUCTION

The use of magnetic resonance imaging (MRI) for target delineation in prostate cancer radiotherapy is widespread due to the superior soft tissue contrast of MRI compared to computed tomography (CT). In a conventional radiotherapy workflow for prostate, CT, and MRI are used in combination by registering the images into a common frame of reference.

Prostate external beam radiotherapy workflows where CT is excluded and solely based on MRI, referred to as an MRI‐only radiotherapy workflow, have now been introduced into the clinic.^1^, ^2^ Systematic uncertainties such as image registration errors between CT and MRI could thereby be eliminated.^3^, ^4^ The impact on target and organ at risk delineation due to anatomical changes between CT and MRI examination, such as bladder and rectum filling, could also be avoided.

The Hounsfield units of the tissues in an MRI‐only radiotherapy workflow are calculated from the MRI‐images and the resulting images are referred to as a synthetic CT (sCT). Currently, two commercial solutions for prostate sCT generation exist, Philips MRCAT and Spectronic MriPlanner.^5^, ^6^ Both solutions have been independently validated and have been or are being used in clinical studies.^1^, ^7^, ^8^ Multiple other solutions for generating a sCT have been presented and were recently reviewed.^9^

An MRI‐only radiotherapy workflow introduces several challenges. One, which will be investigated in this paper, is identification of gold fiducial markers (GFM), inserted into the prostate for target positioning. The GFM has high electron density and will exhibit increased X‐ray attenuation, generating streak artefacts in the CT‐images, mainly caused by inaccurate beam‐hardening correction in the CT‐image reconstruction.^10^ The identification of GFM in CT‐images, in which they cannot be mistaken for calcifications or bleeding, is therefore a straightforward process.^11^

In T2‐weighted (T2w) MRI‐images, GFM, calcifications and bleeding will have similar signal behavior and be depicted as signal voids.^11^, ^12^, ^13^, ^14^, ^15^ This makes differentiating between these objects a challenging task using solely MRI‐images in an MRI‐only radiotherapy workflow.

A common method for identifying GFM using MRI‐images alone is to exploit the difference in magnetic susceptibility between the surrounding tissue and GFM.^5^, ^11^, ^12^, ^14^, ^15^, ^16^ An increased sensitivity to susceptibility effects can be achieved by using gradient echo based MRI sequences.^17^ The resulting shape and size of the signal void from the GFM will not only be dependent on the nature of the MRI sequence and acquisition parameters, but also on the shape and orientation of the GFM.^16^, ^18^

In the previous study, the use of magnetic resonance multi‐echo gradient echo images for GFM identification was suggested. In a human observation study with four observers and 40 patients the sensitivity, specificity, and accuracy of GFM identification in T2w target delineation MRI‐images were determined to be 98%, 94%, and 97%, respectively.^11^ This is similar to human observation studies using other MRI acquisition techniques, which had a detection accuracy between 93% and 98%.^2^, ^12^, ^14^, ^15^, ^19^ For a safe and reliable clinical implementation of an MRI‐only radiotherapy workflow, the GFM identification method should ideally have a detection accuracy of 100%. None of the human observation studies for GFM identification available in the literature have, to the best of our knowledge, reached this detection accuracy. In previous studies, describing clinical workflows for MRI‐only radiotherapy of the prostate, CT was still used for identifying GFM or to differentiate permanent brachytherapy seeds from GFM.^1^, ^2^ Several ways to increase the redundancy of the GFM identification procedure, thereby increasing the identification accuracy, were recently proposed.^19^ The need for improved and cost‐effective GFM identification methods therefore seems evident.

While awaiting improved MR‐based methods for GFM identification when CT is not available, a complementary method to confirm correct MRI‐based identification of GFM could be used. C‐arm X‐ray imaging is often used in the clinic, including ours, to validate a successful GFM implantation. We suggest that the GFM position information from the X‐ray images could be used to differentiate GFM from other objects in the MRI‐images. No additional imaging or changes to the workflow would then be required to validate the GFM identification.

The usefulness of this information relies on the assumption of negligible GFM migration between GFM insertion and imaging of the patient for prostate radiotherapy treatment planning purposes. Mean marker migration has previously been reported to be 0.8 (daily)–1.2 mm (over the entire treatment course).^20^, ^21^

The aim of this work was to show the feasibility of using C‐Arm X‐ray images from the GFM implantation procedures to confirm the GFM identification performed using MRI‐images in an MRI‐only prostate radiotherapy workflow.

## 2 METHODS

### 2.A Patient selection and gold fiducial marker implantation

The first 16 patients in an ongoing MRI‐only prostate radiotherapy study named MR‐PROTECT (MR‐only Prostate RadiOTherapy Excluding CT), representing all data available at the time, were selected for investigation. The patients were prescribed 39 fractions of 2 Gy over 8 weeks. Mean weight (n = 16) was 85.1 ± 10.5 kg [62.0–106.0 kg] and mean age 71.1 ± 5.0 yr [60.0–81.0 yr]. Ten patients underwent ultrasound guided transperineal prostatic implantation of GFM, performed by three different oncologists. Six patients underwent ultrasound guided transrectal implantation, performed by one oncologist. The same type of GFM was used for both implantation methods. One objective, during GFM implantation, was to place the GFM in different areas of the prostate. This could help avoid GFM overlap on orthogonal kilovoltage (kV)‐images, used for patient positioning. This configuration created internal GFM distances of around 2–3 cm, depending on the size of the prostate. No CT data from these patients were used in this study.

As negligible GFM migration was a crucial assumption for the method to work it was of importance to study the migration of the specific GFM type used. To assess the possible impact of GFM migration, a different patient cohort from a conventional CT‐based prostate radiotherapy workflow was selected for investigation. This patient cohort consisted of 33 patients who all underwent ultrasound guided transperineal prostatic implantation of GFM, performed by two different oncologists. One patient was excluded due to loss of GFM and one patient was excluded due to two GFM being inserted to close to each other. Mean weight (n = 31) was 84.9 ± 10.9 kg [62.0–108.0 kg] and mean age 72.9 ± 4.8 yr [60.0–81.0 yr]. No MRI or sCT data from these patients were used in this study.

The GFM consisted of three in‐house produced inferior–superior long axis‐oriented cylinder‐shaped gold objects (length 5.0 mm and diameter 1.0 mm) and were implanted 2 weeks prior to MRI‐ or CT‐imaging. Choice of GFM type was according to the clinic's standard. The study was approved by the regional ethics board with diary number 2013/742, complemented by diary number 2016/801.

### 2.B Imaging and identification of gold fiducial markers

**Figure 1:**
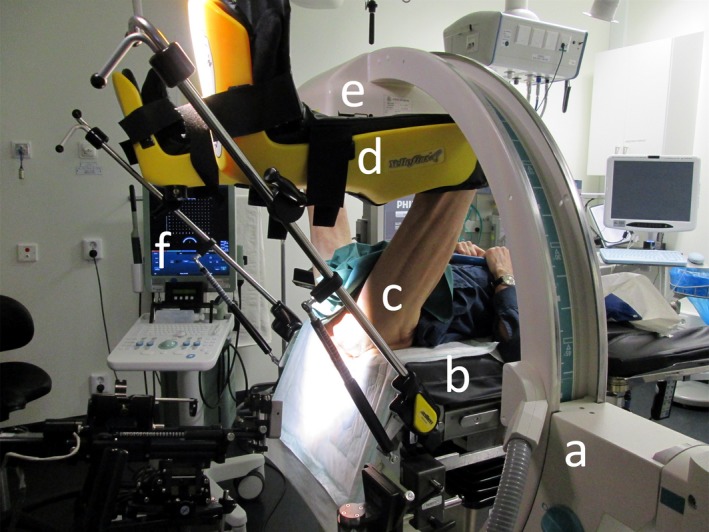
C‐arm X‐ray patient positioning. The patient (c) was placed in a lithotomy position during the transperineal ultrasound (f) guided implantation of the GFM. The legs were fixated using a leg support (d). The C‐Arm X‐ray system (a), with the X‐ray detector (e), was placed in a zero degree angle with respect to the patient table (b) to acquire a posterior‐anterior X‐ray for a successful GFM implantation verification.

**Figure 2:**
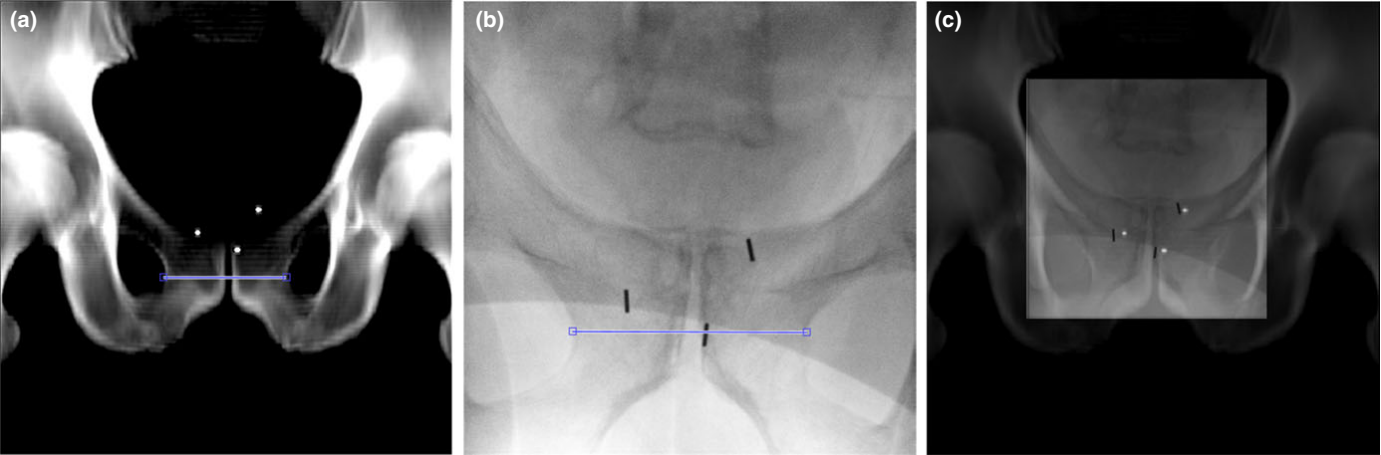
DRR‐ and CkV‐image. Anterior‐posterior DRR‐image generated from sCT with burned in synthetic markers (a), mirrored posterior‐anterior CkV‐image acquired in connection to GFM implantation (b). The CkV‐image scaling was performed by measuring a horizontal distance from left to right over the pubic symphysis in the DRR‐ and CkV‐image (line in a and b). After the CkV‐image was rescaled to the geometry of the DRR, it was manually registered (translation only) using the GFM as a visual aid and overlaid (c).

In connection to the GFM implantation procedure, one posterior‐anterior (PA) X‐ray image was acquired from a portable Ziehm Vision FD Vario 3D C‐Arm X‐Ray system (Ziehm Imaging, ZiehmNetPort, Nuremberg, Germany, software version 5.22) with peak kilovoltage output 60–110 kVp, tube current 6–16 mA, rectangular FOV‐, and 5 mm aluminum filter setting). The PA X‐ray image was, after image acquisition, oriented by the X‐ray system as an AP‐image by left‐right mirroring. All patients undergoing transperineal implantation were imaged in a lithotomy position with the legs placed in a leg support (Fig. 1). For transrectal implantation, the patients were positioned on their side during the implantation and imaged in a supine position with their legs stretched out. The C‐Arm rotation was manually adjusted to a zero degree angle with respect to the patient table, indicated by an analogue protractor scale on the C‐arm (Fig. 1). The actual C‐arm angle used was recorded in the image DICOM header. The C‐arm X‐ray image is hereby referred to as the CkV‐image. The GFM were visualized as low signal intensity objects in the image (Fig. 2[b]).

The transversal sCT‐images used in the MR‐PROTECT study were created from large field of view turbo spin echo T2w MRI‐images with a scan slice thickness of 2.5 mm and 0.7 mm × 0.6 mm in‐plane scan resolution using the conversion software Spectronic MriPlanner version 1.1.2 (Spectronic Medical, Helsingborg, Sweden). The software and the MRI imaging protocol were recently validated against CT.^22^, ^23^ In the MR‐PROTECT study, the center of mass (CoM), for each GFM in each patient, was manually identified in the sCT geometry using Eclipse Treatment Planning system version 13.6 (Varian Medical Systems, Palo Alto, CA, USA). The identification of GFM in the T2w MRI‐images, i.e., sCT geometry, was aided by multi‐echo gradient echo MRI‐images.^11^ The determined GFM CoM coordinates were used by Spectronic MriPlanner to burn in synthetic markers onto the sCT‐images (slice thickness 2.5 mm, 0.4 mm × 0.3 mm in‐plane resolution). The synthetic markers were depicted as round 2‐D high intensity objects, defined in one slice each, with a diameter of 4 mm. Correct positioning of the synthetic markers in the sCT‐images, created in the MR‐PROTECT study, was verified prior to the treatment.

CT‐images were, for the patients in the conventional CT‐based prostate radiotherapy workflow (to assess GFM migration), acquired with a Siemens Somatom Definition AS+ (Siemens Healthcare, Forchheim, Germany), slice thickness 3 mm, reconstructed diameter 500 mm, reconstructed in plane resolution 0.98 mm × 0.98 mm, peak kilovoltage output 120 kVp, exposure time 500 ms and tube current 213–660 mA using CareDose.

Anterior‐posterior (AP) oriented digitally reconstructed radiographs (DRR) with isotropic pixel resolution using a 512 × 512 matrix were created from CT and sCT at a gantry angle of 0 degrees using the Eclipse Treatment Planning System. The DRR from the CT and sCT is hereby referred to as CTDRR and sCTDRR, respectively.

### Image processing and analysis

**Figure 3:**
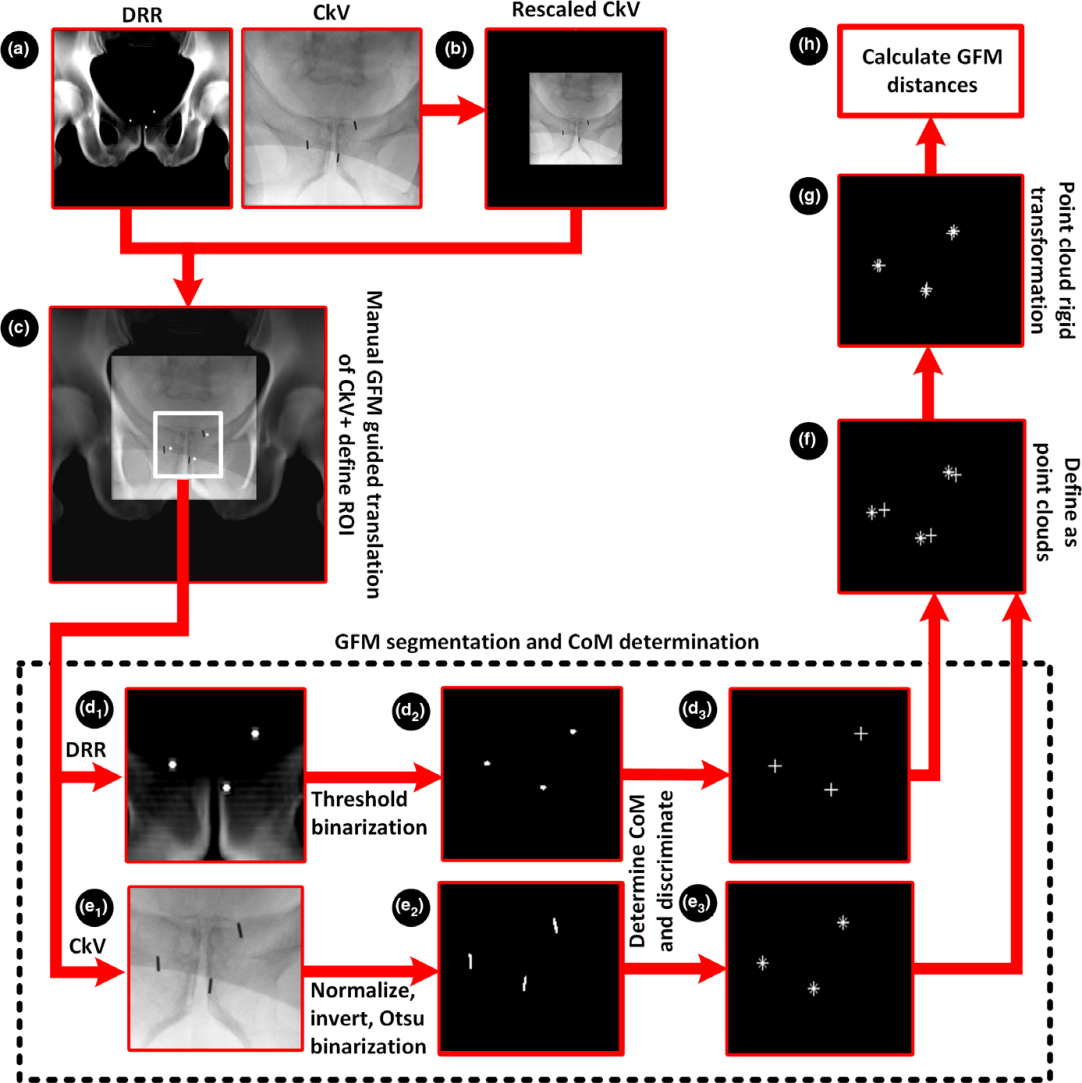
Workflow for the proposed method (a) sCTDRR‐ and CkV‐image was acquired, (b) the CkV‐image was rescaled to the image resolution of the sCTDRR‐image, (c) the rescaled CkV‐image was visually and manually overlaid onto the sCTDRR‐image and a rectangular ROI around the GFM was defined and used as an image mask, (d_1‐2_) the masked sCTDRR‐image was binarized using a threshold chosen to suppress non‐GFM objects, (e_1‐2_) the masked scaled CkV‐image was normalized, inverted and binarized using Otsu's segmentation method,^25^ (d_3_) and (e_3_) the 2‐D connected components in the binarized sCTDRR‐ and CkV‐image were identified, a discrimination of the identified connected components was performed and the CoM of the GFM was determined, (f) the GFM CoM coordinates in the sCTDRR‐ and CkV‐image defined two point clouds, (g) a rigid transformation between the point clouds was calculated, (h) the distances between each GFM in the registered point clouds were calculated. The same workflow (a‐h) was applied to CTDRR to assess GFM migration.

Image processing and analysis were performed using an in‐house developed MATLAB program with a graphical user interface (version R2017a, Mathworks Inc., Natick, MA, USA). The steps described below were applied to both sCTDRR, CTDRR, and the corresponding CkV‐image (Figs. 2 and 3).

Generation of a common geometric frame of reference between the DRR and CkV‐images was necessary due to the different image modality origin. The DICOM information from the CkV‐image did not contain information about the spatial resolution of the image. For each patient, the CkV‐image was scaled to the image resolution of the DRR with an in‐house developed method. The scaling was performed by measuring a horizontal distance from left to right over the pubic symphysis in the DRR with start and stop points defined in the left‐right interface between the obturator foramen and the body of pubic bone (Fig. 2). The corresponding distance in the CkV‐image was identified and image rescaling was performed using bicubic interpolation.^24^ To quantify the uncertainty in the determination of the image scale factor, horizontal distance measurements in the DRR‐ and CkV‐image were, by one user, repeated 15 times each for three random patients, selected from the sCTDRR cohort. The largest coefficient of variation (CV) in the image scale factor among the three patients was considered to be a measure of scale factor uncertainty. All distance measurements were performed with a resolution superior to the inherent image resolution.

The rescaled CkV‐image was visually overlaid onto the DRR‐image using a translational manual registration using the GFM as visual guidance. This was performed in order to supply the point cloud registration (see later steps) with information on point‐coupling for the three GFM in the DRR‐ and CkV‐image.

The GFM was automatically identified in the DRR‐image by first masking a rectangular area positioned around the central part of the pelvis containing the GFM. The masked DRR‐image was binarized using a threshold chosen to suppress non‐GFM objects. The 2‐D connected components with a connectivity of at least eight pixels were identified. A discrimination of the identified connected components using prior knowledge of the upper and lower GFM object size in the image was performed and the CoM of the GFM was determined (Fig. 3).

The GFM was automatically identified in the scaled CkV‐image by first masking the image using the previously defined DRR‐mask (expecting similar image geometry). The masked scaled CkV‐image was normalized, inverted, and binarized using Otsu's segmentation method.^25^ The selection of GFM and the determination of the CoM were performed using the same techniques as for the DRR‐image described above. (Fig. 3).

For each DRR‐ and CkV‐image, a point cloud was defined from the GFM CoM coordinates. A point cloud is a set of data points containing spatial coordinates. Each point cloud in this study had three data points containing the three GFM CoM 2‐D coordinates. A rigid transformation between the two point clouds was calculated using an iterative closest point algorithm.^26^ The transformation was applied to the GFM CoM coordinates for the CkV‐image. Visual inspection of the GFM CoM point‐clouds was performed after the point‐cloud transformation (Fig. 3).

The absolute total difference in CoM between each of the GFM in the DRR‐image and the rigidly transformed GFM CoM coordinates for the scaled CkV‐image was calculated as a total distance and a distance in the directions left‐right and inferior‐superior. The absolute total distances calculated for patients in the conventional CT‐based prostate radiotherapy workflow was considered to be a measure of GFM migration.

To assess if there was a difference in the measured absolute total distance between the GFM in the sCTDRR and CkV compared to CTDRR and CkV, a nonparametric two‐sided independent Mann‐Whitney U‐test^27^ with a 5% significance level was used.

## 3 RESULTS

Using the proposed method together with visual inspection, all GFM in the sCTDRR cohort were confirmed to have been previously correctly identified. The mean absolute total difference in CoM displacement between the GFM for sCTDRR‐ and CkV‐image was 1.7 mm (1 SD = ±1.4 mm) (Table 1). Patient 11 in the sCTDRR cohort had the largest absolute distance difference, observed in one of the GFM, located in the inferior‐superior direction (6.3 mm). This distance difference was larger than three standard deviations from the mean of 1.7 mm. This raised a concern regarding a potential erroneous identification of one GFM and the cause of this outlier was investigated. It could, without any additional imaging, be concluded that it was due to the large marker migration (see Section 2.B).

**Table 1:** Mean absolute difference in CoM between the GFM in the DRR‐image and the rigidly registered scaled CkV‐image. Data are presented for patients in an MRI‐only prostate radiotherapy workflow (sCTDRR vs CkV). GFM migration data are presented for patients in a conventional CT‐based prostate radiotherapy workflow (CTDRR vs CkV). The absolute difference in the directions left‐right, inferior‐superior, and in total is denoted by ∆X, ∆Y, and ∆Total

	Mean (mm)	SD (mm)	Median (mm)	Minimum (mm)	Maximum(mm)
CTDRR vs CkV (n = 31)					
∆X	0.9	0.7	0.8	0.0	3.2
∆Y	0.6	0.5	0.4	0.0	2.1
∆Total	1.2	0.7	1.1	0.0	3.2
sCTDRR vs CkV (n = 16)					
∆X	0.8	0.7	0.7	0.0	2.8
∆Y	1.3	1.4	0.7	0.0	6.3
∆Total	1.7	1.4	1.2	0.1	6.3

The mean GFM migration was determined from the CTDRR cohort as the mean absolute total distance difference between the CTDRR and CkV image (1.2 mm (1SD = ±0.7 mm)). The mean and standard deviation for the absolute total distance difference were smaller for the CTDRR cohort compared to the sCTDRR cohort (Table 1).

The mean image scale factor with one SD and CV for the three random patients, randomly selected from the sCTDRR cohort, was 4.35 ± 0.043 [4.28 4.42] CV = 1.0% (n = 15), 3.56 ± 0.024 [3.52 3.60] CV = 0.7% (n = 15), and 3.66 ± 0.032 [3.62 3.72] CV = 0.9% (n = 15). The uncertainty determination for the image scale factor was therefore estimated to be 1%.

No statistically significant difference in the measured total distances of the GFM in the sCTDRR and CkV compared to CTDRR and CkV could be detected (*P* = 0.37). The mean absolute total distance in the sCTDRR cohort (1.7 mm) could therefore not be separated from possible GFM migration effects. The distance of 1.7 mm was therefore considered as a small and acceptable distance in the proposed method.

Three patients in the sCTDRR cohort had deviations of one, three, and five degrees, respectively, from the desired C‐Arm zero degree angle setting, which led to non‐perfect PA imaging projections. For the CTDRR cohort, four patients had a deviation of one degree. The angle deviations from zero degrees were believed to be the cause of human error.

## 4 DISCUSSION

The developed method demonstrated the feasibility of using a single CkV‐image from the fiducial implantation procedure to confirm the GFM identification performed in an MRI‐only prostate radiotherapy workflow. The method was dependent on calculating and evaluating the distance between each of the GFM in the CkV‐image and a DRR‐image created from sCT‐images in the MRI‐only radiotherapy workflow.

The spatial accuracy for manual and automatic GFM identification methods, solely using MRI‐images, has previously been reported to be sufficient but, due to prostatic calcifications and bleeding, the detection accuracy has been insufficient.^1^, ^11^, ^12^, ^13^, ^14^, ^15^, ^19^ From the results of this study and with discussed uncertainties in mind, our proposed method can detect if errors in the GFM identification process have occurred. This routine could be applied to both manual and automatic GFM identification frameworks.

To assess the impact of GFM migration, specific to the GFM type and imaging schedule used in our clinic, the method was applied to DRR‐ and CkV‐images for 31 patients, included in a conventional CT‐based prostate radiotherapy workflow. A statistical difference between the accuracy with respect to GFM displacement in the MRI‐only workflow and conventional CT‐based workflow could not be detected. The mean absolute total distance in the sCTDRR cohort (1.7 mm) could therefore not be separated from possible GFM migration effects.

GFM migration effects could explain a major part of the mean absolute total distance difference measured in the sCTDRR cohort (1.7 mm). Further, it has been shown that the mean geometric accuracy to which 3–5 mm cylindrical GFM (oriented parallel to the magnetic field) can be identified in spin echo based target delineation MRI‐images is around 1 mm (1 SD = 1 mm).^11^, ^18^ As the sCT‐images inherit the geometry from the full field of view T2w MRI‐images, the same geometric accuracy can be expected for the synthetic markers in the sCT‐images. The synthetic markers in the sCTDRR‐images, created from the sCT‐images, will therefore also be affected. With these discussion points in mind and a GFM inter‐distance of about 2–3 cm, a mean absolute total distance of 1.7 mm between the sCTDRR‐ and CkV‐image could be regarded as small enough for the method to be used as a QA tool for GFM identification.

The measured mean absolute total distance difference in the CTDRR cohort for marker migration assessment is believed to consist of GFM migration in combination with inherent uncertainties in the current evaluation method. These uncertainties could arise due to measurement errors in the image scaling involving manual selection of start and stop points, the elevated patient leg position for CkV‐imaging or a non‐perfect CkV‐image PA projection of the patient anatomy (three patients deviated from a zero degree C‐arm angle setting). The same inherent uncertainties were assumed to exist in the sCTDRR cohort. Measured distances from the CTDRR cohort were, however, similar compared to previously reported mean values of 0.8–1.2 mm GFM migration.^20^, ^21^ This suggests that the inherent uncertainties in the proposed evaluation method could be considered minor and the dominating effect in the measurements of the CTDRR cohort was due to GFM migration.

Implantation of the GFM for the patients in the sCTDRR cohort was performed using both transrectal and transperineal procedures while patients in the CTDRR cohort was only subjected to transperineal based implantation procedure. The difference in the implantation procedures is not expected to affect GFM migration^28^, ^29^ and therefore not the results in this study either.

The rescaling of the CkV‐image to the geometry of the sCTDRR cohort depended on defining a distance within a bone structure (Fig. 2). A sCT‐image might not depict those bones with the same amount of details and geometric fidelity as a CT‐image. The scaling of the CkV‐image in the sCTDRR cohort would then be affected. The uncertainty for the image scale factor was measured and estimated to be as small as 1%. Further, the creation of sCTDRR was not optimized as the default clinic DRR creation settings for CT was used (not sCT adapted). It is possible that these factors have contributed to the measurement of absolute total difference in GFM distances for the sCTDRR cohort. These contributions are, however, considered minor.

Recently, CT was still used for identification of GFM in clinical workflows for MRI‐only radiotherapy of the prostate.^1^, ^2^ With the method proposed in our study, the use of CT would not be necessary. The proposed method could potentially also be used to mitigate the recently suggested need for redundant processes in GFM identification.^19^

If the assumption of negligible GFM migration between GFM insertion and MRI imaging of the patient was not fulfilled, the spatial GFM position correlation for a GFM, imaged at different time points with different modalities, would be weaker. This would make it harder to detect an incorrectly identified GFM using the proposed method. Patient 11 in the sCTDRR cohort had a large (6.3 mm) absolute distance difference for one of the GFM. Through review of the previously acquired multi‐echo gradient echo MRI‐images^11^ or using RT kV‐images from the first treatment fraction, it could be concluded that the deviation was due to an actual GFM migration in the inferior‐superior direction. The large migration was believed to be a result of a complication during the implantation procedure.

Another situation with imperfect GFM implant geometry could occur if multiple GFM overlap in the projection image. One of the objectives during GFM implantation is to avoid such a scenario. Unfortunately, one patient in the CTDRR‐cohort was excluded due to inability to separate overlapping GFM. This limitation can be resolved with a lateral CkV‐image.

The rigid point cloud registration used in this method depended on an iterative closest point (ICP) algorithm which used minimization of the mean squared distance as optimization metric. If all GFM migrated the same distance in the same direction, the algorithm would be unable to differentiate between GFM migration and prostate movement. Migration would then not be detected. The algorithm also assumes no GFM to be stationary in its position. This implies that if only one GFM migrated and the others did not, the migration distance would be underestimated. These scenarios are, however, not considered likely to occur and are not regarded as potential issues.

The rigid point cloud registration accounted for in‐plane prostate rotation but the image information in the single PA CkV‐image limited the ability to detect and correct arbitrary prostate rotation between GFM implantation and MRI‐imaging. This did, however, not seem to constitute a notable problem for the proposed method.

The measured absolute difference in GFM CoM between the DRR‐ and CkV‐image for the two cohorts was similar in the left‐right component. The largest directional difference and variation were found in the inferior‐superior component for the sCTDRR cohort (Table 1). The dominating factor for this is believed to be an effect of the prior manual identification of the GFM CoM in the transversal large field of view T2w MRI‐image (originating from the MR‐PROTECT study) — as the GFM CoM was forced to be positioned in an existing transversal slice (not in between any slices). This limited the spatial resolution in determining the CoM in slice direction (patient inferior‐superior). This limitation propagated to an uncertainty of the GFM CoM in the inferior‐superior direction of the AP sCTDRR. A reduced slice thickness of the T2w MRI‐image (assuming unchanged image quality) would yield an improved spatial accuracy for the manual GFM identification.

The determination of the CoM of the GFM in the CTDRR used image interpolation between the image slices, was not dependent on any prior manual identification steps, and was not subjected to the above problem with limited spatial resolution (other than slice thickness). It is believed that this effect largely contributed to the numerical difference in the measured mean absolute total difference and uncertainties for the two cohorts.

The mean absolute distance difference between the GFM for sCTDRR‐ and CkV‐image was 1.7 mm (1 SD = ±1.4 mm). The mean value with additional two standard deviations adds up to 4.5 mm. Given the experimental conditions discussed and the results of this study, the authors therefore suggest that a non‐acceptable distance difference between the GFM in the sCTDRR‐ and CkV‐image for the proposed method is greater than 5 mm.

In the event of detecting a non‐acceptable distance difference between the sCTDRR‐ and CkV‐image the user should reevaluate the MRI‐images to see if any calcifications or other similar objects exist in the vicinity of the determined GFM signal void. If not, actual GFM migration can be concluded. A multi‐echo gradient echo MRI acquisition technique can facilitate this task^11^ and this approach proved to be successful for the assessment of the large migration in one of the GFM for patient 11 in the sCTDRR cohort.

The image information from a PA CkV‐image did not enable a full 3‐D verification of the GFM positions and this constituted a limitation in the proposed method. However, in our suggested prostate MRI‐only radiotherapy workflow, a last verification step is performed at the first radiotherapy fraction where orthogonal kV‐images are acquired. Future improvements would be to use a radiopaque ruler placed on the patient during CkV imaging — this could eliminate the need for anatomy based image scaling. A lateral CkV‐image should also be added to the implantation procedure CkV‐image acquisition. The additional patient anatomy information would then enable a full 3‐D verification of the GFM positions.

## 5 CONCLUSIONS

To confirm GFM identification in MRI‐images, performed in an MRI‐only prostate radiotherapy workflow, a C‐arm X‐ray image acquired from the GFM implantation procedure could be used. GFM migration is present but does not constitute a problem for the proposed method. The method can therefore be considered suitable for the task.

## CONFLICTS OF INTEREST

The authors declare no conflict of interest.
